# Applying strategies from libertarian paternalism to decision making for prostate specific antigen (PSA) screening

**DOI:** 10.1186/1471-2407-11-148

**Published:** 2011-04-21

**Authors:** David C Wheeler, Konrad M Szymanski, Amanda Black, David E Nelson

**Affiliations:** 1 Occupational and Environmental Epidemiology Branch, Division of Cancer Epidemiology and Genetics, National Cancer Institute, Bethesda, MD 20892, USA; 2 Division of Urology, McGill University Health Centre, Montreal, QC, Canada; 3 Epidemiology and Biostatistics Program, Division of Cancer Epidemiology and Genetics, National Cancer Institute, Bethesda, MD 20892, USA; 4 Cancer Prevention Fellowship Program Branch, Center for Cancer Training, National Cancer Institute, Bethesda, MD 20892, USA

## Abstract

**Background:**

Despite the recent publication of results from two randomized clinical trials, prostate specific antigen (PSA) screening for prostate cancer remains a controversial issue. There is lack of agreement across studies that PSA screening significantly reduces prostate cancer mortality. In spite of these facts, the widespread use of PSA testing in the United States leads to overdetection and overtreatment of clinically indolent prostate cancer, and its associated harms of incontinence and impotence.

**Discussion:**

Given the inconclusive results from clinical trials and incongruent PSA screening guidelines, the decision to screen for prostate cancer with PSA testing is an uncertain one for patients and health care providers. Screening guidelines from some health organizations recommend an informed decision making (IDM) or shared decision making (SDM) approach for deciding on PSA screening. These approaches aim to empower patients to choose among the available options by making them active participants in the decision making process. By increasing involvement of patients in the clinical decision-making process, IDM/SDM places more of the responsibility for a complex decision on the patient. Research suggests, however, that patients are not well-informed of the harms and benefits associated with prostate cancer screening and are also subject to an assortment of biases, emotion, fears, and irrational thought that interferes with making an informed decision. In response, the IDM/SDM approaches can be augmented with strategies from the philosophy of libertarian paternalism (LP) to improve decision making. LP uses the insights of behavioural economics to help people better make better choices. Some of the main strategies of LP applicable to PSA decision making are a default decision rule, framing of decision aids, and timing of the decision. In this paper, we propose that applying strategies from libertarian paternalism can help with PSA screening decision-making.

**Summary:**

Our proposal to augment IDM and SDM approaches with libertarian paternalism strategies is intended to guide patients toward a better decision about testing while maintaining personal freedom of choice. While PSA screening remains controversial and evidence conflicting, a libertarian-paternalism influenced approach to decision making can help prevent the overdiagnosis and overtreatment of prostate cancer.

## Background

Screening for prostate cancer using prostate specific antigen (PSA) has become widespread despite the controversy surrounding the practice. For men, prostate cancer is the most commonly diagnosed cancer in the US and the second leading cause of cancer death. In 2010, approximately 217,730 men were diagnosed with prostate cancer and 32,050 men died, illustrating the public health burden of this disease and the need for the identification of effective methods to reduce prostate cancer mortality [1].

Unfortunately, there is a lack of conclusive evidence of the value of PSA screening in the reduction of mortality; two randomized controlled PSA screening trials, one in Europe and the other in the US, recently reported disparate results for the effect of PSA testing on prostate cancer mortality [2,3]. The European study reported a 20% relative reduction in prostate cancer mortality with screening while the American trial reported no mortality difference. The hope that these studies would provide further clarity and direction for PSA screening did not materialize. A more recently published population-based screening trial reported a cumulative relative risk reduction of death as a result of prostate cancer of 50% in the screening group, however the risk of overdiagnosis was still substantial [4]. In addition to the inconsistent results of the clinical trials, there is disagreement in PSA screening guidelines from major health organizations [5-8].

The fact that PSA testing leads to overdiagnosis and overtreatment of prostate cancer and subsequent harms, such as incontinence and impotence, adds to the controversy about PSA screening. Given the ongoing controversy, the responsibility for the decision to screen a man for prostate cancer is a key issue in the PSA debate. In this paper, we discuss the challenges of decision making for PSA screening for prostate cancer and argue for augmenting the existing approaches of informed decision making (IDM) and shared decision making (SDM) with strategies from libertarian paternalism (LP) to improve decision making.

## Discussion

### Existing decision-making approaches

Traditionally, medical practice has been a paternalistic system, with the health care provider telling the patient what to do and making the final decisions regarding screening or treatment. More recently, informed decision making and the related concept of shared decision making are increasingly being advocated, and several health organizations recommend that a patient be well informed about the risks and benefits of screening with PSA, and that he discusses his screening decision with his health care provider [5]. Both approaches of IDM and SDM aim to empower patients to choose among the available options by making them active participants in the decision making process [9,10]. In the IDM approach, the patient is presented with all the information pertinent to making a decision and then assumes final authority for the decision. In the SDM approach, the patient is provided with all the relevant information and works with the health care provider to reach a decision that reflects the health preference of the patient [11]. Traditionally, the information relevant to the clinical decision has been conveyed verbally to the patient by the health care provider.

An increasingly important part of the IDM and SDM approaches is decision aids, which are designed to consistently deliver comprehensive and objective information to the patient. Decision aids, such as an informational videotape or pamphlet, have been shown to increase patient knowledge about prostate cancer and its management options [12-15]. Information relevant to the clinical decision conveyed verbally to the patient by the health care provider can be supplemented with the use of a decision aid [16]. Several studies have shown that a significantly lower proportion of patients choose PSA testing among those who were given decision aids to assist in informed consent [17,18] and SDM [13,19] compared to those in control groups. The American Cancer Society, a long-time advocate of IDM, recommends in its most recent guidelines the use of decision aids [8]. Decision aids also have a potentially important role in malpractice defence by enabling practitioners to document that the screening decision of a patient was an informed one based on reviewing a decision aid [20,21].

Proponents of the IDM and SDM approaches argue that one of the important benefits of these approaches is that the final decision for screening or treatment is more in agreement with the values and best interests of the patient than in the traditional paternalist approach [8,22]. While this is certainly true, by increasing involvement of patients in the clinical decision-making process, IDM/SDM places more of the responsibility and pressure for a complex decision on the patient.

### Challenges to decision-making

In practice, several challenges to decision making for PSA screening remain. Various prostate cancer screening guidelines recommend a balanced discussion between the health care provider and the patient about risks and benefits of PSA screening prior to screening [7,8]. However, research suggests that these discussions are not consistently taking place [16,23], that patients are often not properly educated about screening [23,24], and that individuals face biases that may interfere with making an informed decision [25-27].

Clinicians have been slow to adopt the IDM and SDM approaches and as a result they are currently underutilized. Practical issues, such as the time required for a detailed discussion of clinical options with patients, have limited the adoption of SDM or IDM [16]. Clinicians under heavy time constraints may find it quicker and easier to order a PSA test than to carefully counsel the patient. Fear of malpractice litigation or financial incentives inherent in the health care system have also likely hindered the adoption of IDM/SDM by clinicians. In a recent random survey of 375 men aged 40 and older who had either discussed PSA testing or had a PSA test in the previous two years, only 55% of men were asked their preference for screening [23]. In a small random sample of the medical charts of elderly patients in the VA study [28], only 4% of ordered PSA tests were reported to be requested by patients.

Another barrier to decision making is that patients often lack knowledge of prostate cancer and screening tools and are not fully informed of the risks and benefits of screening [24]. A recent survey demonstrates a lack of patient knowledge about PSA testing and prostate cancer, as 48% of respondents failed to answer correctly any one of three knowledge questions about prostate cancer screening [23]. Health care providers may not emphasize in a discussion all the information that patients might consider important [29], and they may also present information in an unbalanced way. In one study, the health care provider more often emphasized benefits of PSA screening (71%) than its harms (32%) [23]. Another survey found that physicians placed less value on providing information to patients than did patients [30]. Physicians may also overestimate the level of patient knowledge about the disease and treatment options [29]. As a result, patients may not get all the information they need from their physician to make a decision. SDM and IDM approaches are only fully effective when patients are truly well informed [31,32]. The increasing use of decision aids should help to educate patients about the risks and benefits of screening and also alleviate the time burden on clinicians and increase the utilization of IDM.

While IDM/SDM approaches are promising for allowing a patient to make a decision that is concordant with their values and preferences, barriers to rational thought could influence the preferences expressed by patients. Traditional economic decision-making models assume that individuals are consistent, rational decision-makers. However, evidence from studies in psychology and behavioural economics shows that individuals are often not good judges of what would improve their well-being, and they frequently make uninformed or suboptimal choices regarding their own welfare [25-27,33]. Individuals are subject to an assortment of biases (e.g. discounting, omission, status quo, loss aversion [25,26,34,35]), and emotion (e.g., fear, anxiety), which can generate irrational thoughts that interfere with making informed choices. Status quo bias will lead a patient to adhere to the path of least resistance even if a superior option exists [34]. Status quo bias can also be described as a tendency for inertia and going along with the default option. Omission bias entails the preference for a more harmful act of omission (not taking action) over a less harmful act of commission (taking action) [25]. If a patient's health care provider always recommends PSA testing and portrays it as an automatic course of action, then status quo bias or omission can lead them to follow this course of action even if it is not in the patient's best interest. The idea of discounting future harms such as incontinence or impotency for the current benefit of peace of mind from a low PSA value can explain a decision for an immediate PSA test. A strong emotional fear of cancer and the desire for an urgent peace of mind can lead to a rash decision about screening when it is presented as an immediate option. Patients may also have difficulty assessing probabilities, and as a result are often overly optimistic about their own outcome when faced with the probabilities of survival with treatment options [36] and overestimate their risk of disease. For example, it has been reported that some men overestimate their lifetime risk of developing and dying from prostate cancer [37].

In summary, considering the current challenges to decision making on PSA screening, there are opportunities to apply new strategies to the existing decision-making process. Given the widespread promotion of PSA screening through the media [28,31], by some health-related organizations and providers, and through social networks [38], the general enthusiasm for screening in the United States [39], personal fears and overestimation of one's own longevity [40], and the individual and system-wide factors influencing health care providers, patients are more likely to undergo PSA testing than not, resulting in unnecessary treatment and harms.

### Libertarian paternalist strategies for PSA screening

Libertarian paternalism is a philosophy that uses the insights of behavioural economics to help people better make better choices [26]. A libertarian paternalistic plan is aware of the biases that may hinder a rational decision by individuals and leads to the design of the choice architecture to overcome some of these biases [33]. Major distinguishing features of libertarian paternalism that are relevant for PSA screening decision making include a default decision rule, the framing of the information influencing the decision, and the timing of the decision. Strategies based on these aspects of LP can be applied to the IDM/SDM approaches to improve decision making about PSA screening.

#### Default decision

In an LP plan or program, there is a default decision rule. The rule states the default option or decision, which is the decision automatically made if an individual does not opt to make a decision of their own. As example, a default rule could be that every eligible person is automatically entered into a program (plan A), or conversely, that eligible persons must elect to enter the program on their own (plan B). In an LP-based approach, an individual is free to go against the default setting by making his or her own decision. If an individual goes against the default when it is an automatic enrolment (plan A), they opt-out of the program. Conversely, if the individual decides against the default when it is an optional enrolment (plan B), they opt-in to the program. In other words, deciding against a default option of no required action for participation is called opt-out, whereas going against inertia to participate is called opt-in. Therefore, the default decision rule in plan A corresponds to an opt-out strategy and the rule in plan B corresponds to an opt-in strategy. Cleary, what is considered as an opt-in or opt-out strategy depends on the default option.

For a more tangible example, a company retirement savings plan that has as its default option automatic enrolment of employees into saving a fixed percent of their income would require employees to opt out of the plan if they did not want to save for retirement. A more common employee retirement savings plan would have a default of no saving and would require employees to opt in to the plan to save through the employer. In a libertarian paternalistic plan, the default is carefully selected by the choice architect with the best interest of the individual in mind, and is ideally based on consistent research evidence. The choice of the default option is important because individuals must overcome status quo bias in order to select a non-default option.

The choice of the default option has been shown to be quite influential in both non-clinical and clinical settings. For example, changing the default option from optional enrolment (opt-in) to automatic enrolment (opt-out) for employment-based retirement savings plans has greatly increased participation rates and likely increased the long-term financial well-being for many people [41,42]. In another example, people were almost twice as willing to be organ donors when the default strategy was opt-out (i.e., people would be considered as organ donors unless they specifically requested not to be donors) instead of opt-in [43].

Substantially higher levels of recruitment of patients for health care research have been seen when the default strategy is opt-out [44]. Similarly, health survey participation and response rates are substantially higher when the default strategy is opt-out compared with opt-in [45,46]. Default policies have also been shown to improve health care quality. A large body of research has demonstrated that standing orders for adult immunizations in hospitals and other settings, for which opting out of receiving pneumococcal or other vaccines is the default, greatly increases immunization rates [47]. A default policy to remove urinary catheters after 72 hours has been recommended to reduce the number of nosocomial infections [48].

#### Framing

Framing is another important strategy of libertarian paternalism that can be influential in decision making. Framing is the expression of logically equivalent information in different ways [49], and can be expressed in either negative or positive terms. For example, negative framing would state that a patient has a 20% chance of dying from a treatment, while positive framing would state the same patient has an 80% chance of surviving with the treatment.

An individual may be more likely to select a choice if it is presented with positive framing [27]. Also when using framing, emphasizing the potential loss from selecting an action can be more powerful than emphasizing the potential gain from that action due to individuals' tendency to loss aversion [25]. In other words, deciding on not taking an action and missing out on a benefit would be considered more favourable than deciding to take an action and then be harmed as a result. In practice, framing has been shown to influence personal choice when deciding on medical procedures [27,33,50]. In one example, when patients were asked to choose between two equally efficacious medications, 57% chose the medication whose benefit was expressed in relative terms while only 15% chose the medication whose benefit was expressed in absolute terms [50].

#### Timing

Timing is another LP strategy that can be used when designing a choice system to produce better decisions. LP discourages rash decisions and builds in a sufficient amount of time before an individual makes a decision, which can encourage more rational thought and consideration of the available options and how they confirm to the values of the individual. This time allows for what Sunstein and Thaler [26] refer to as "sober reflection" of one's decision.

In health care settings, including a time delay should limit the number of decisions based on an immediate, powerful, and emotional fear of illness or a lack of understanding of the harmful side-effects of potential treatment. Timing can also be used to influence action or inaction through status quo bias, as the duration of the time delay between presentation of an option and the opportunity to select it could affect levels of inertia. A time delay for reflection combined with an opt-in system could increase inertia. For example, a substantial required time delay before deciding on a particularly risky procedure could decrease the likelihood of selecting that option.

#### Strategies in practice

The LP strategies of a default decision, framing, and timing can be applied to decision making for PSA screening in the IDM/SDM approaches. Adding a system default decision that minimizes harm may help reduce overdiagnosis and overtreatment.

The default decision we propose is no PSA screening, that is, an opt-in system. This default is appropriate for minimizing harm when benefits are uncertain, which is the current situation with PSA screening with an absence of definitive evidence that PSA screening reduces prostate cancer mortality and that the benefits outweigh the risks. Clearly, this default would be modified should this situation change based on further research and level of scientific consensus.

It is noteworthy that the default decision is for both patients and health care providers using the LP-influenced approach. This does not mean that clinicians could not recommend screening, but that the default decision would be to not screen. This is consistent with IDM/SDM in that it forces an information transfer to the patient or a discussion with the clinician before a decision to screen occurs. While the default decision of no PSA screening can be overridden, its presence would likely reduce the extent of testing.

This approach requires the use of decision aids and a consent form for PSA screening to document that a patient was informed before making a decision. A form could also be presented for the patient to sign at the time informational materials are presented to document receiving the decision aids. These documents could be important for potential malpractice defence. The decision aids would be designed to provide an independent and evidence-based presentation of the positives and negatives of PSA screening, expectantly avoiding patient and physician bias and overcoming the failure of information transmission due to clinician time constraints.

Framing could be used in the decision aids and consent form to emphasize either the benefits of screening (possible early detection of cancer) or the harms (possible incontinence and impotence) of screening and the cascade of procedures resulting from a false positive test. A more negatively framed statement for prostate cancer screening using PCA and digital rectal examination would be that out of every 10 abnormal test results, 7 men will not have prostate cancer [14]. The more positively framed statement of the same results would be that out of every 10 abnormal test results, 3 men will have cancer. Emphasizing the potential loss in urinary function and sexual function over the potential gain of reassurance of no cancer or early detection of cancer could influence patient decision making. The nature of the framing used when presenting information for a choice should depend on the current state of evidence. With the current situation of a lack of evidence of the benefits of PSA screening, the harms should be emphasized more strongly through framing.

Timing would be used in this approach to PSA screening decision making to provide the patient time to review the decision aids and consider the benefits and harms of screening. After reviewing the informational materials and carefully considering the options over a reasonable amount of time, say 1 week, the patient who elects screening would then be required to sign a written consent form. This process requires the patient to opt-in to testing only after a time delay that allows for sober reflection, and would be similar to the process used to obtain informed consent from patients for medical procedures. The additional time for sober reflection afforded by the consent form and the effort needed to overcome inertia would likely decrease the number of PSA tests that are requested based on an immediate, powerful, and emotional fear of illness or lack of understanding of the harmful side-effects of potential treatment for prostate cancer.

While a screening approach that adopts libertarian paternalistic strategies could help reduce overdiagnosis and overtreatment from PSA screening, there are several issues to consider for implementation of such an approach. One issue is who is to take the responsibility for implementing the approach and making the important decisions needed for implementation. The default option must be selected using the current state of scientific evidence. Given a lack of consensus about the screening decision, the default could be based on the most common recommendation in the screening guidelines from major health organizations or, perhaps more justifiably, on the recommendation from the health organization with the most rigorous review process for determining a guideline. The specific set of information to be presented in the decision aids and consent form must also be decided upon. For this, a panel of clinical and communication experts could be convened to review and recommend decision aids.

It could be argued that what we propose raises ethical concerns because of the default choice of no PSA screening. In other words, LP imposes certain values and some individuals might not receive a test from which they might benefit. Similar arguments have been raised regarding HIV testing of patients [51], where the default strategy is opt-in and there are concerns about unforeseen harms for individuals. We argue, however, that the approach with LP strategies described is more explicit and honest about underlying values than the current situation. Our core values are simply that a cancer screening test should not be performed without clear and convincing research evidence and consensus that it reduces mortality, and the fundamental medical principle of first do no harm.

## Summary

PSA screening has been over-utilized in the United States as a preventative measure for prostate cancer, and in many instances has likely caused more harm than benefit. Given the current lack of conclusive evidence of the ability of PSA screening to differentiate between clinically significant and indolent localized prostate cancer, and to reduce prostate cancer mortality, a more structured and conservative decision-making approach for screening would be beneficial to minimize harming individuals unnecessarily. Libertarian paternalism has been used to guide individuals to making better decisions by designing choice systems that acknowledge barriers to thoughtful, well-reasoned decisions. Our proposed application of key libertarian paternalism strategies to augment the current IDM and SDM decision-making approaches to PSA screening is intended to provide patients with the current state of knowledge on screening, and to protect them from making rash, uninformed decisions with the potential for significant harm while maintaining personal freedom of choice.

We hope definite evidence will emerge and that improved methods may eventually be able to differentiate between high-grade prostate cancer and clinically harmless disease, rendering a libertarian paternalistic approach for PSA screening unnecessary. In the interim however, while PSA screening remains controversial and evidence conflicting, this approach to decision-making can help prevent the overdiagnosis and overtreatment of prostate cancer.

## Competing interests

The authors declare that they have no competing interests.

## Authors' contributions

DCW drafted the manuscript. All authors contributed to and approved the final manuscript.

## Pre-publication history

The pre-publication history for this paper can be accessed here:

http://www.biomedcentral.com/1471-2407/11/148/prepub
